# Osseointegration Effect of Micro-Nano Implants Loaded With Kaempferol in Osteoporotic Rats

**DOI:** 10.3389/fbioe.2022.842014

**Published:** 2022-02-23

**Authors:** Anyue Wang, Wenhong Yuan, Yu Song, Yanjun Zang, Yanling Yu

**Affiliations:** ^1^ Department of Stomatology, School of Stomatology of Qingdao University, Qingdao, China; ^2^ Qingdao Municipal Hospital, Qingdao, China; ^3^ Qingdao Stomatological Hospital Affiliated to Qingdao University, Qingdao, China

**Keywords:** Osteoporosis, implants, micro-nano, Kaempferol, Osseointegration, chitosan, gelatin

## Abstract

**Objective:** To investigate the effect of osseointegration of kaempferol loaded on the surface of micro-nanomorphic implants in ovariectomized rats.

**Methods:** Titanium flakes were polished to obtain the PT group, anodized and acid-etched to obtain the NT and WNT groups, loaded with kaempferol to obtain the KNT and KWNT groups, and spin-coated on chitosan-gelatin composite film to obtain the KNT-CG and KWNT-CG groups. *In vitro* experiments were performed to observe the physicochemical properties of the titanium tablets in each group through scanning electron microscopy and contact angle experiments. The cytotoxicity and drug release pattern were observed using CCK-8 and drug release assays. An osteoporosis rat model was established. Pure titanium implants were divided into PT, NT, WNT, KNT-CG, and KWNT-CG groups after the same treatment and used in the *in vivo* experiments and then implanted in the femur of mice in each group. After 4 weeks, all samples were collected for toluidine blue staining, micro-computed tomography scanning, and bone morphometry analysis to evaluate their osteogenic properties.

**Results:** According to scanning electron microscopy, the surface of the titanium flakes had a micro-nano morphology in the WNT group and the KNT and KWNT groups were functionally loaded with kaempferol. In CCK-8 and drug release experiments, the loaded kaempferol and gelatin composite membranes showed no significant toxic effects on cells. The drug release time in the KNT-CG and KWNT-CG groups was significantly longer than that in the KNT and KWNT groups, with the release time in the KWNT-CG group reaching 15 days. *In vivo* experiments micro-computed tomography and bone morphometry analysis showed that the osteoporosis model had been successfully constructed. The bone volume fraction around the implant increased. Toluidine blue staining showed new bone formation and a significantly increased number of bone trabeculae.

**Conclusion:** Kaempferol micro-nanocomposite coating improved the osseointegration ability of implants in osteoporotic rats.

## Introduction

Osteoporosis (OP) is divided into two major categories: primary and secondary. Postmenopausal OP in women is the most common form of primary OP. As the world population increases, OP has become an important medical issue (Teitelbaum, 2010; Rachner et al., 2011).

Additionally, the rate of missing teeth in middle-aged and elderly people is gradually increasing. Currently, titanium dental implants are widely used because they can achieve osseointegration by crossing the area of cancellous bone, inducing the cytomes and bone trabeculae of fibroblasts and osteoblasts to reach the titanium surface and form a biomechanical bond with the interfacial interface of the body’s fine weave via a chemical reaction (Noguerol et al., 2006; Turkyilmaz et al., 2008). However, in the case of OP, the clinical presentation is often characterized by alveolar ridge resorption and reduced alveolar bone density due to lower local and overall bone density. This results in a prolonged bone healing time after implant placement as well as a decreased osseointegration rate and a lower initial stability of the implant. Thus OP patients have a poor osseointegration profile (Merheb et al., 2016). In order to improve the success rate of implant surgery in patients with osteoporosis, the following methods are currently used for treatment: bone extrusion technique, systemic drug treatment and implant surface treatment. Since the bone extrusion technique is not easy to master, excessive extrusion can easily cause implant failure. The systemic drug treatment will bring inevitable side effects, so the main focus of this group is on implant surface treatment. Numerous studies have shown that micro-nano treatment of the implant surface can improve the osseointegration effect. Numerous studies have shown that micro-nano treatment of the implant surface can be performed to improve the osseointegration effect, but the results remain unsatisfactory (Milinkovic et al., 2012; Sulka et al., 2013; Zhao et al., 2020; Yang et al., 2021). Micro-nanostructures have the effect of promoting osteoblast adhesion, while drug can be loaded on their surface as drug delivery carriers. However, the drug release time of micro-nanostructures alone is short for long-term osteogenesis, so membranous materials that can prolong the drug release time are considered to cover the surface. Both chitosan and gelatin have good biocompatibility and biodegradability. Wang et al. found that chitosan exhibited higher drug loading and better sustained release performance after forming composites with nanostructures (Wang et al., 2008). Javanbakht et al. demonstrated the sustained release function of gelatin and its high stability for long-term drug delivery through *in vivo* drug tests, meanwhile the results of cytotoxicity experiments showed that it has low toxicity to cells. Therefore, this experiment proposes to load both on the material surface (Javanbakht et al., 2019).

Chinese herbal medicine has been used in China for more than 2000 years to treat OP and in recent years has gradually attracted worldwide attention because of its low side effects (Yza et al., 2021). Modern pharmacological studies have shown that kaempferol, an extract of many herbal medicines, can promote osteoblast differentiation (Trivedi et al., 2008; Kumar et al., 2010; Guo et al., 2012) and inhibit osteoclast activity (Wattel et al., 2003; Kim et al., 2018). Therefore, it may be possible to form a composite structure by loading kaempferol on the surface of micro-nanomorphic materials to exert joint effects. In this study, by simulating bilateral ovariectomy in rats, a postmenopausal female OP model was established to investigate the effects of combining micro-nanoformations with kaempferol on bone metabolism neonatal bone tissue, bone density, and bone trabecular microstructure in de-ovaried rats.

## Materials and Methods

### Experimental Animals

Thirty-six 3-month-old specific pathogen-free-grade female Sprague-Dawley rats were purchased from Jinan Pengyue Experimental Animal Breeding Company (Shandong, China). All animal-related experiments were performed using the protocols approved by our institute (The Ethics Committee of Qingdao Stomatological Hospital Affiliated to Qingdao University, 2019KQYX025). The body weight of the rats was 240 ± 20 g. Two rats were housed in each metabolic cage under standard conditions and used in the experiment after 1 week of acclimatization feeding.

### Experimental Methods

#### Functionalized Morphology Construction and Material Characterization

We sanded 14 × 1-mm pure titanium flakes samples (Dongguan Shengyue Precision Hardware Products Co., Guangdong, China) with 600-, 1,000-, 3,000-, 5,000- and 7000-mesh sandpaper until their surfaces were smooth and mirror-like. The samples were ultrasonically cleaned with acetone, anhydrous ethanol, and deionized water for 15 min each in turn to remove surface impurities and then dried naturally to obtain the smooth (PT) group samples. PT samples was placed in 1.25 wt% hydrofluoric acid (HF) solution for acid etching to form a micron-level morphology. Further experiments were conducted to reduce the surface diameter, and the specimens with a micron-level morphology were placed in 1.5 wt% HF to prepare the nanoscale morphology by anodic oxidation. The specimens were cleaned by ultrasonic cleaning with deionized water for 15 min and dried under natural air. The micro-nano group (WNT) with micron-nano coexistence morphology and nano group (NT) with pure nanoscale morphology were obtained.

#### Loading of Kaempferol on the Material Surface

The randomly selected NT and WNT group were placed in 10 μM (Kim et al., 2016) kaempferol (Beijing Solebro Technology Co., Beijing, China, purity 99.62%, molecular weight 286.24 Da) in anhydrous ethanol, and then placed in a vacuum drying oven and evacuated. The vacuum pump was turned off after 15 min, and the samples were left under vacuum for 3 days, followed by natural air-drying for 1 day. Residual drug on the surface was removed by washing with phosphate-buffered saline (PBS) to obtain the kaempferol nano-group (KNT) and kaempferol micro-nano-group (KWNT).

#### Sample Surface Covered With Chitosan/Gelatin Composite Film

First, a mixture of 1% formic acid solution and 2 g of chitosan was added to the formic acid solution to prepare a 2% chitosan formic acid solution by stirring with a magnetic rotor for 2 h at a speed of 1,000 r/min at 37°C. Next, 10 mg of gelatin was dissolved in deionized water and incubated at room temperature until the gelatin was completely dissolved. A homogeneous 1% gelatin solution was prepared by stirring in a water bath at 60°C for 2 h (Hao et al., 2021; Zheng et al., 2021). The KNT and KWNT samples were placed in a vacuum rotary coater set to a speed of 4,000 r/min. Chitosan solution was added slowly with a dropper onto the sample surface and rotated to coat the sample for 40 s. The configured gelatin solution was sucked again in the same manner, and two layers of each solution were applied alternately. Dried and ready for use (Wang et al., 2021). Finally, kaempferol nano plus film set (KNT-CG) and kaempferol micro nano plus film set (KWNT-CG) were produced.

#### Scanning Electron Microscopy Observation of Sample Surface Morphology

The surface morphology and characteristics of the samples were observed using scanning electron microscopy (SEM; Thermo Fisher Scientific, Waltham, MA, United States) of the PT, NT, WNT, KNT, KWNT, KNT-CG, KWNT-CG groups. Samples from each group were fixed with conductive adhesive, observed, and photographed.

#### Contact Angle Detection

Five samples were randomly selected from each group, and the contact angle of each sample was tested with a static contact angle tester (DSA25E, Kruss, Germany). Deionized water (10 µL) was added dropwise to the sample surface at room temperature and left for 40 s. After the water droplets were stabilized, images of the water droplet spreading on the sample surface were obtained with a camera. Each sample was evaluated three times and the average value was taken. Software was used for detection and analysis. The results were 90° < contact angle <180° for hydrophobic and 0° < contact angle <90° for hydrophilic.

#### Detection of Early Release Pattern of Kaempferol

A solution of kaempferol at 28.6 mg/L in ethanol was prepared as the master solution and diluted into a series of solutions with concentrations of 1.43, 2.86, 4.29, 5.57, 8.58, and 11.44 mg/L. Anhydrous ethanol was used as a control, and kaempferol was detected at a wavelength of 366 nm. The samples were sequentially injected into a UV spectrophotometer (UV, Shanghai Metash Instruments Co., China) to evaluate the peak areas.

The samples were injected into the UV spectrophotometer, and the peak areas were recorded. Samples from the KNT, KWNT, KNT-CG, and KWNT-CG groups were added to a 24-well plate, soaked dropwise with 3 ml of PBS, pH 7.4, and heated in a water bath and set at 100 r/min and 37°C. Every second day, 500 µL of soaking solution was collected by aspiration, and 500 µL of fresh PBS was added to continue soaking. The concentration of kaempferol in the aspirated solution was detected by UV detection until the concentration was no longer detectable, i.e., all kaempferol had been released into the PBS. The average release dose and load of all samples was calculated at the same time point. A cumulative release curve was drawn (He et al., 2021).

#### CCK-8 Assay for Cytotoxicity

A 10 μM kaempferol solution was prepared and CCK-8 was used to detect the cytotoxicity of kaempferol towards MC3T3-E1 cells after binding to the micro-nanomorphs. Samples from each group were disinfection and placed at the bottom of 24-well plates, the cells were inoculated at a density of 5 × 10^4^ cells/mL, and the plates were incubated for 1, 3, 5, and 7 days at 37°C and 5% CO_2_ in an incubator. The samples were then washed with PBS, and 0.25% trypsin was added for digestion. Then transferred to 96-well plate and incubated in an incubator. After 24 h, 10 μL CCK-8 was added to each well, and the plate was incubated at 37°C for 4 h to induce nasal production. The optical density of each well at 450 nm was measured using an enzyme immunoassay analyzer (BioTek Instruments, Winooski, VT, United States), and cytotoxicity was considered to exist when the relative cell viability was below 70% (ISO, 2009).

Both the drug release and cytotoxicity assays demonstrated that the KNT-CG and KWNT-CG groups had better performance than the KNT and KWNT groups. Therefore, the latter two groups were excluded from subsequent animal experiments.

#### Animal Experiments

Twenty-eight Sprague-Dawley rats were randomly divided into two groups: 24 rats in the experimental group underwent bilateral ovariectomy (OVX); in the control group of four rats, only a small portion of adipose tissue around the ovaries was removed (sham operated). Sprague-Dawley rats were acclimatized for 1 week and anesthetized by intraperitoneal injection. The skin, fascia, and muscles were separated bluntly layer-by-layer, and the ovaries were removed from the experimental group after ligating the fallopian tubes and adjacent vascular tissues around the ovaries with a No.0 surgical wire; a small amount of adipose tissue around the ovaries was removed from the control group. After the operation, the rats moved normally and had free access to food. Penicillin was injected intramuscularly at 250,000 IU/kg for three consecutive days to prevent infection. Four weeks later, four rats in each from the experimental and control groups were sacrificed using an overdose of anesthesia. Both femurs from each rat were separated, and the surrounding attached soft tissues were removed to preserve the integrity of the femur as much as possible. The femurs were completely soaked in 4% paraformaldehyde for micro-CT (μCT-100, Hangzhou Yuebo Biotechnology Co., China) scanning to confirm the generation of the OP model.

**FIGURE 1:**
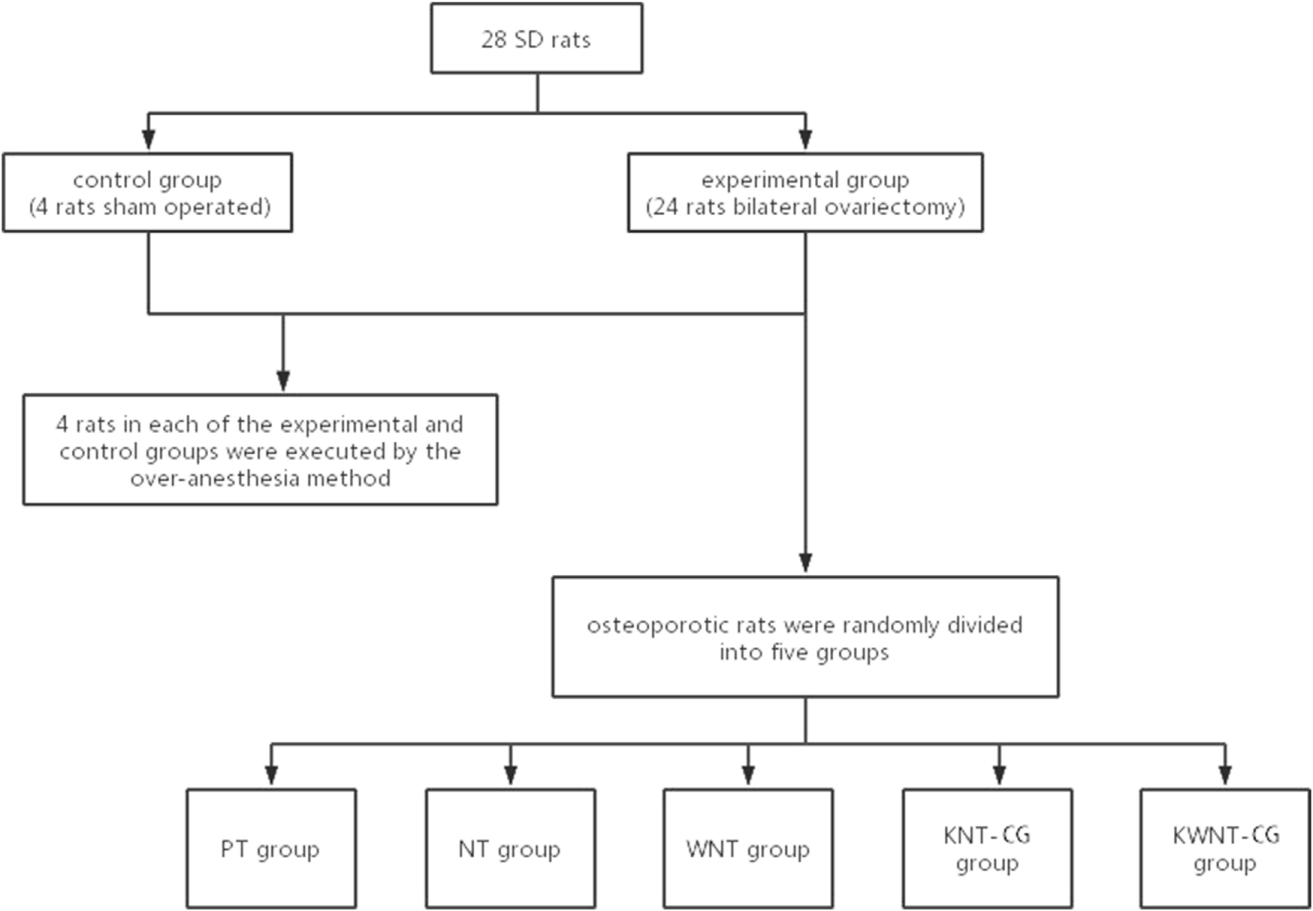
Animal experimental procedures.

After successful modeling, the osteoporotic rats were randomly divided into five groups of four rats each, and the skin was cut at the distal femur bilaterally. The subcutaneous tissue was bluntly separated to reach the femoral epiphysis, and pre-prepared titanium nails from the PT, NT, WNT, KNT-CG, and KWNT-CG groups were implanted (titanium nails were treated in the same manner as titanium pieces). Postoperatively, penicillin was injected intramuscularly for three consecutive days at 250,000 IU/kg to prevent infection. At 4 weeks after nail implantation, the rats were sacrificed through anesthesia overdose to extract the material, and the same method was used as above for subsequent testing. The specific process is shown in Figure 1.

#### Micro-CT Scanning

After 24 h of fixation, the samples were removed, scanning and 3D reconstruction were performed by micro-CT with the implant as the axis and bone spread at around 2 mm to the surrounding bone. The scanning parameters were as follows: voltage 70 kV, current 200 μA, resolution 14.8 μm, and exposure time 300 ms. Bone volume (BV), tissue volume (TV), object surface/volume ratio (BV/TV), trabecular number (Tb.N), trabecular thickness (Tb.Th), trabecular spacing (Tb.Sp) and degree of anisotropy (DA) were measured separately.

#### Toluidine Blue Staining

The grinding slides were prepared using an EXAKT hard tissue cutting and grinding system (300 CP/400CS/AW, EXAKT, Germany) as follows. Fresh tissue specimens were fixed in 40 g/L paraformaldehyde fixative at 4°C for 24–48 h. After fixation, the specimens were dehydrated in ethanol solution and then immersed in anhydrous ethanol and light-curing resin for infiltration. The fully infiltrated tissues were placed in a light-curing embedding machine for polymerization. The tissue blocks were cut to obtain the target sections and polished step-by-step to prepare approximately 50-μm-thick tissue abrasives. Finally, the slices were sealed with neutral resin after toluidine blue staining.

### Statistical Analysis

Paired *t*-tests were performed using SPSS 12.0 software (SPSS, Inc., Chicago, IL, United States), and differences were considered as statistically significant when *p* < 0.05.

## Results

### SEM Observation Results

**FIGURE 2:**
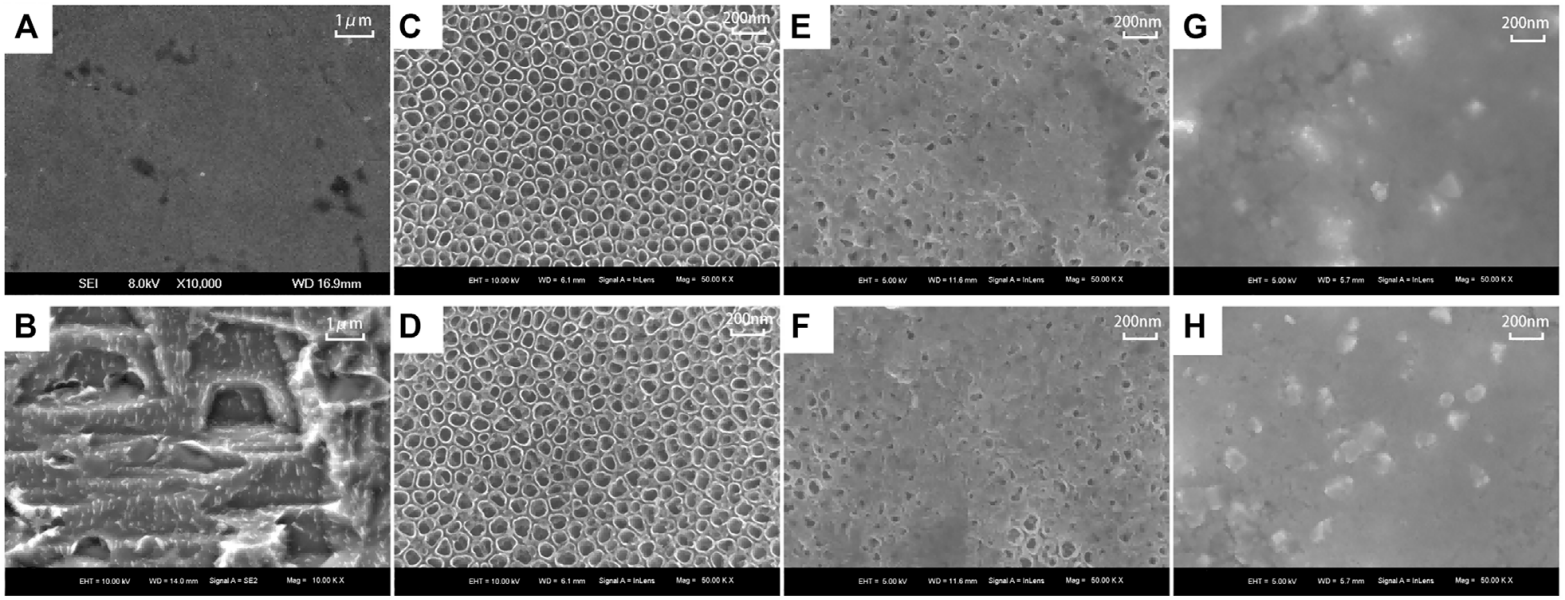
Surface morphology of each titanium sheet under SEM. **(A)**: PT group; **(B)**: Micron structure; **(C)**: NT group; **(D)**: WNT group; **(E)**: KNT group; **(F)**: KWNT group; **(G)**: KNT-CG group; **(H)**: KWNT-CG group.

The surface morphology of titanium flakes in the PT group was smooth with only a few polishing marks (Figure 2A). The surface morphology formed by acid etching was micron-sized and stepped (Figure 2B). The KNT and KWNT groups had a nanoscale and micro-nanoscale morphology surface loaded with kaempferol, most nanotubes orifices were not visible because of drug blockage, and kaempferol was successfully loaded into the nanotubes (Figures 2E,F). The KNT-CG and KWNT-CG groups formed nanotubes with a micro-nanoscale structure after the surface was coated with chitosan and a gelatin film bilayer (Figure 2E). The microstructure was covered, appearing to have a patchy surface (Figures 2G,H).

### Contact Angle Analysis

**FIGURE 3:**
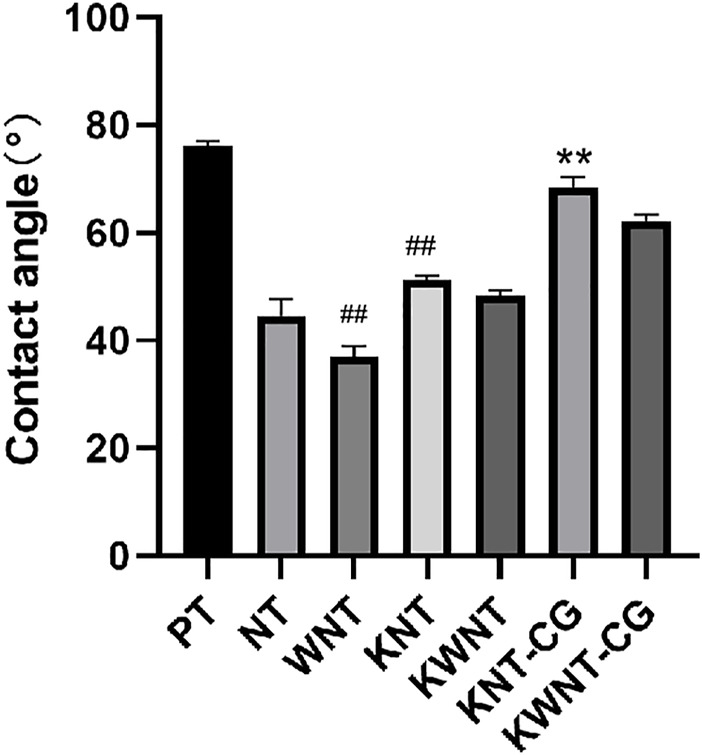
Analysis of the results of contact angle comparison between groups. Bar graphs: Data are presented as mean ± SD. ^#^ represents comparison with NT group, ^##^ *p* < 0.01; * represents comparison with PT group, **p < 0.01.

The contact angles of the seven groups of samples were determined, as shown in Figure 3. The relationship between the surface contact angle of the experimental materials in each group is PT group > KNT-CG group > KWNT-CG group > KNT group > KWNT group > NT group > WNT group. Due to the formation of micron structure and binding to nanotubes enhanced hydrophilicity, the contact angle of the NT group > WNT group (*p* < 0.01). The KNT group showed lower hydrophilicity compared to the NT group because of drug loading (*p* < 0.01). However, since titanium metal was relatively hydrophobic, the KWNT-CG group < PT group (*p* < 0.01).

### CCK-8 Assay Results

**FIGURE 4:**
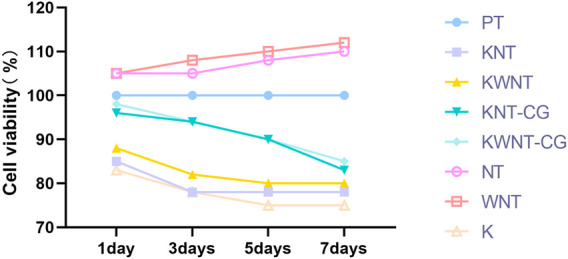
Analysis of CCK-8 results.

The results of the CCK-8 assay are shown in Figure 4; the PT group was used as the control. The cytotoxicity of the KNT and KWNT groups increased from 1 to 3 days because of rapid drug release; the KNT-CG and KWNT-CG groups were coated with chitosan and gelatin membranes, which slowed drug release and reduced cytotoxicity. However, the relative cell viability was greater than 70%, demonstrating that the micro-nanostructures loaded with kaempferol and coated with membranes were less toxic towards cells (Zhou et al., 2020).

### Analysis of Kaempferol Release

#### Establishment of the Standard Curve

**FIGURE 5:**
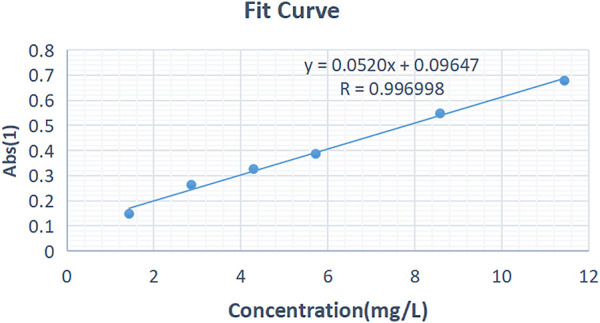
Concentration-absorbance standard curve of kaempferol solution.

The standard curve was drawn using linear regression with the concentration C (mg/L) as the horizontal coordinate and absorbance Abs of the solution as the vertical coordinate. The linear equation was as follows: Y = 0.0520X + 0.0947 (R = 0.996998). A linear relationship was observed between the kaempferol concentration and absorbance in the range of 0.1–15 mg/L, as shown in Figure 5.

#### Determination of Drug Release

**FIGURE 6:**
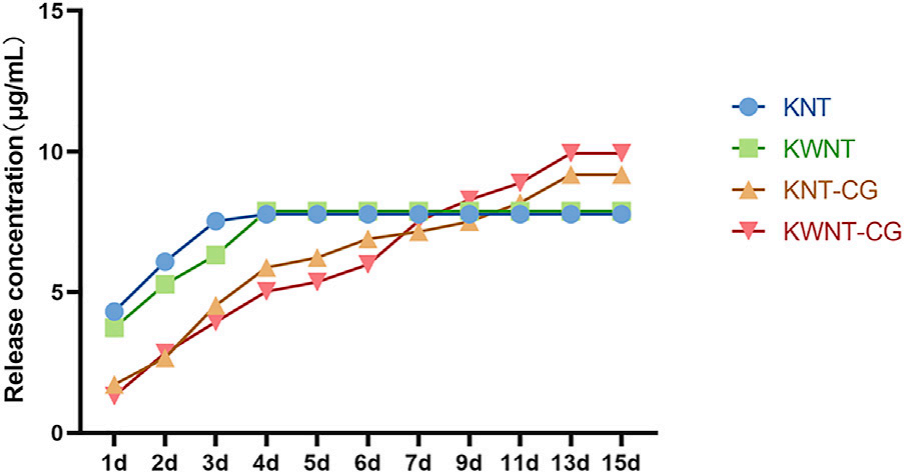
Cumulative release curve of titanium tablets loaded with kaempferol.

Figure 6 shows the cumulative release curves of kaempferol from the four groups of samples within 15 days. The KNT group showed complete drug release in the first 3 days, with the KWNT group exhibiting drug release within 4 days because of its micron structure. After coating with the chitosan and gelatin composite film, the loaded kaempferol in the KNT-CG and KWNT-CG groups showed a longer slow release, and the total amount of drug released exceeded that in the KNT and KWNT groups. The KWNT-CG group showed the highest total drug release.

### Results of Micro-CT Scans

#### Scanning Results of OP Mouse Model

**FIGURE 7:**
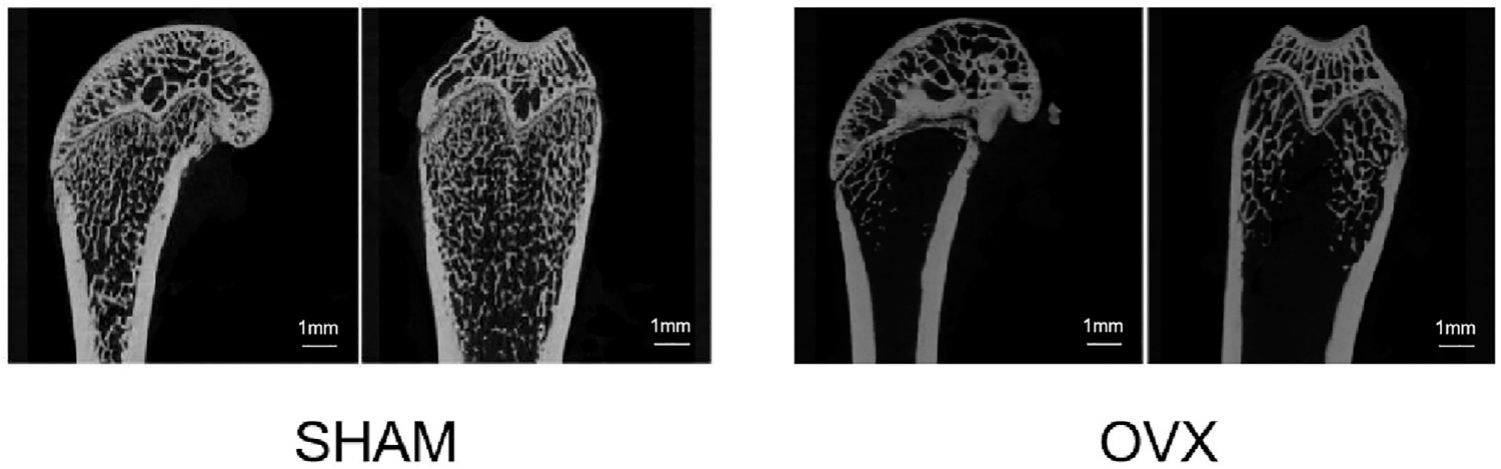
Micro-CT scan section of rat femur. Compared to the sham operation (SHAM) group, the bilateral ovariectomized (OVX) group had reduced bone trabecular density and successful osteoporosis modeling.

Figure 7 shows the micro-CT images of the femur in the sham and OVX groups. Bone density was significantly lower in the OVX group than in the sham group, indicating that the animal model of OP was successfully constructed.

#### Micro-CT Bone Volume and Bone Microstructure Parameters

**FIGURE 8:**
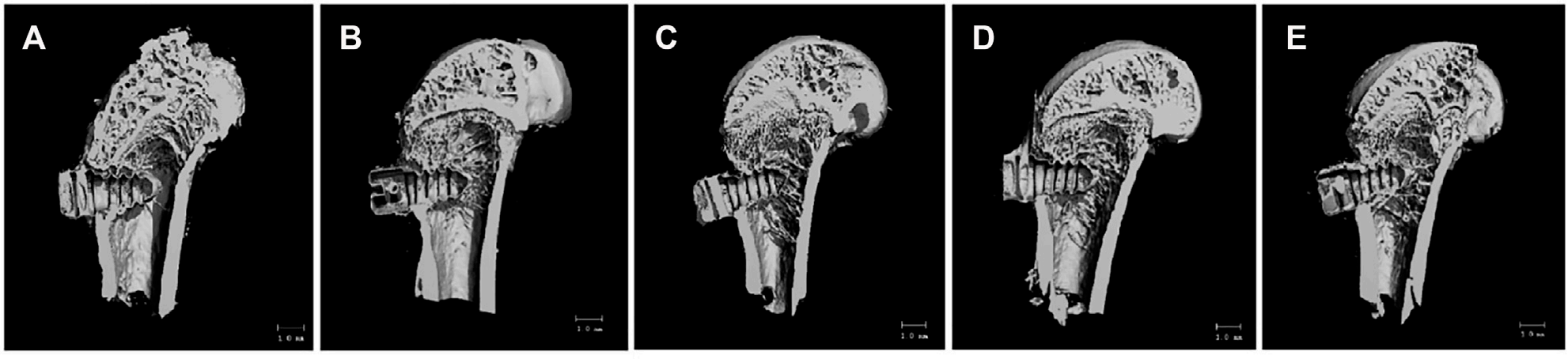
3D Micro-CT scan of rat femur. **(A)**:PT group; **(B)**:NT group; **(C)**: WNT group; **(D)**:KNT-CG group; **(E)**:KWNT-CG group.

**FIGURE 9:**
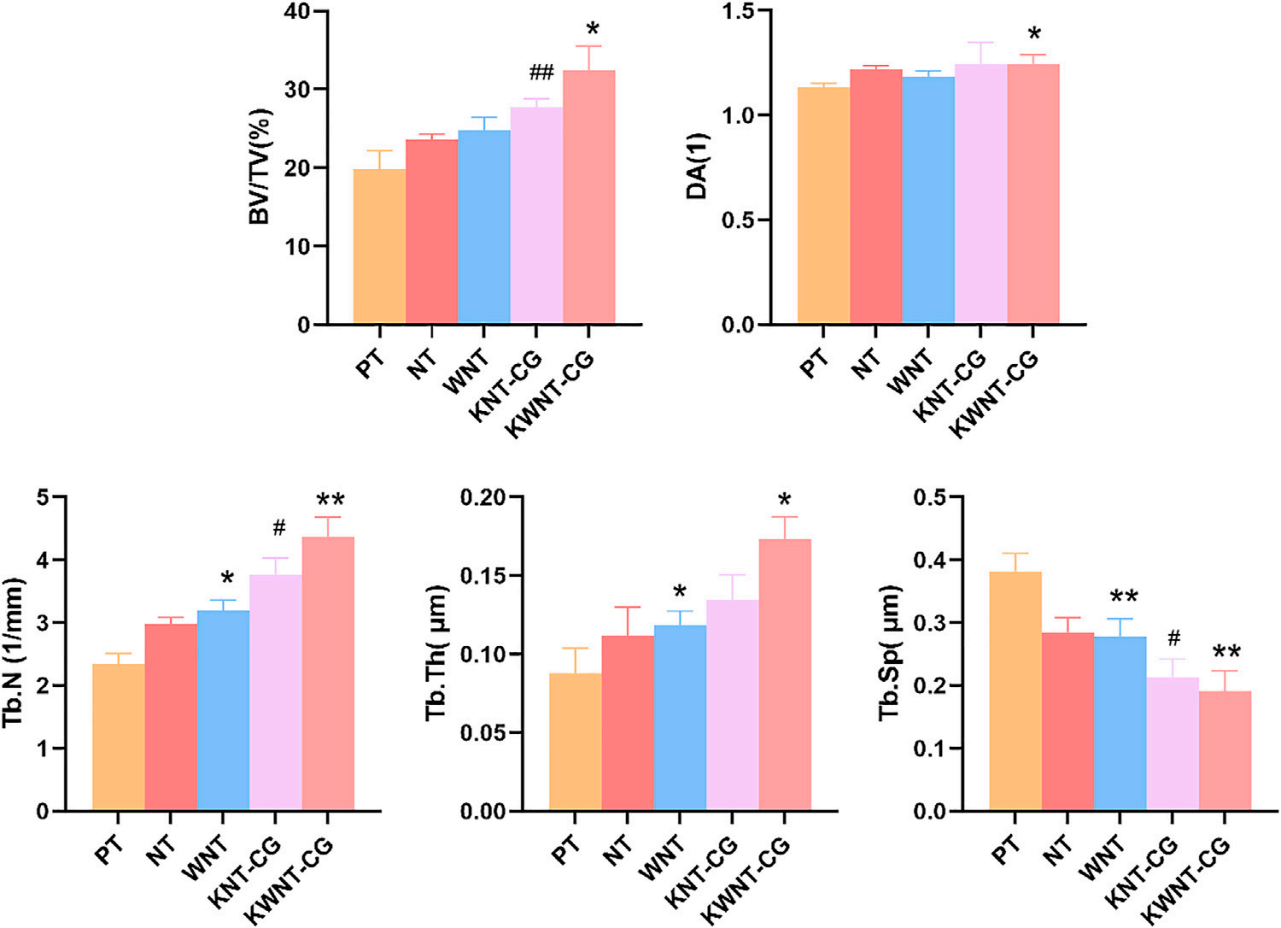
Morphometric analysis of rat femur. BV/TV, bone volume per tissue volume; DA, degree of anisotropy; Tb.N, trabecular number; Tb.Th, trabecular thickness; Tb. Sp, trabecular sepacing. Bar graphs: Data are presented as mean ± SD. ^#^ represents comparison with NT group, ^##^ *p* < 0.01, ^#^ *p* < 0.05; *represents comparison with PT group,**p < 0.01; *p < 0.05.

As shown in Figure 8A, the bone trabecular spaces around the femoral implants of rats in the PT group were larger, withy sparser and more slender bone trabecular structures formed locally. As shown in Figure 8B–E, the number of bone trabeculae was significantly increased, continuity was higher, and separation was reduced, indicating that kaempferol release promoted osseointegration. The best improvement was observed in the KWNT-CG group, where the bone trabeculae were tightly interwoven to form an irregular three-dimensional meshwork resembling a sponge. Bone morphometric analysis was performed (Figure 9). The BV/TV value is the most important parameter reflecting bone remodeling. BV/TV values increased in the KNT-CG group compared with the NT group (*p* < 0.01) and in the KWNT-CG group compared with the PT group (*p* < 0.05). These results indicate the osteogenic effect of micro-nanoconjugated loaded drugs and chitosan-gelatin bilayer. Tb.N, Tb.Th and Tb.Sp are the main indicators for evaluating the spatial morphological structure of bone trabeculae, and each of these values was higher in the WNT group than in the PT group (*p* < 0.01), indicating that the micro-nano structure did promote an increase in the number of bone trabeculae and led to a decrease in their separation. Owing to the loading of the drug and covered with chitosan-gelatin bilayer, the Tb.N and Tb.sp values were higher in the KNT-CG group than in the NT group (*p* < 0.01). The Tb.Sp values were also higher than in the NT group, but there was no statistical difference in Tb.Sp (*p* > 0.05). The DA values in the KWNT-CG group were higher than those in the PT group (*p* < 0.05), indicating better orientation and symmetry of the bone trabeculae. The titanium implants in each experimental group increased the number, thickness, and volume fraction of the bone trabeculae, improving local femoral OP and contributing to osseointegration of the implants.

### Analysis of Toluidine Blue Staining Results

**FIGURE 10:**
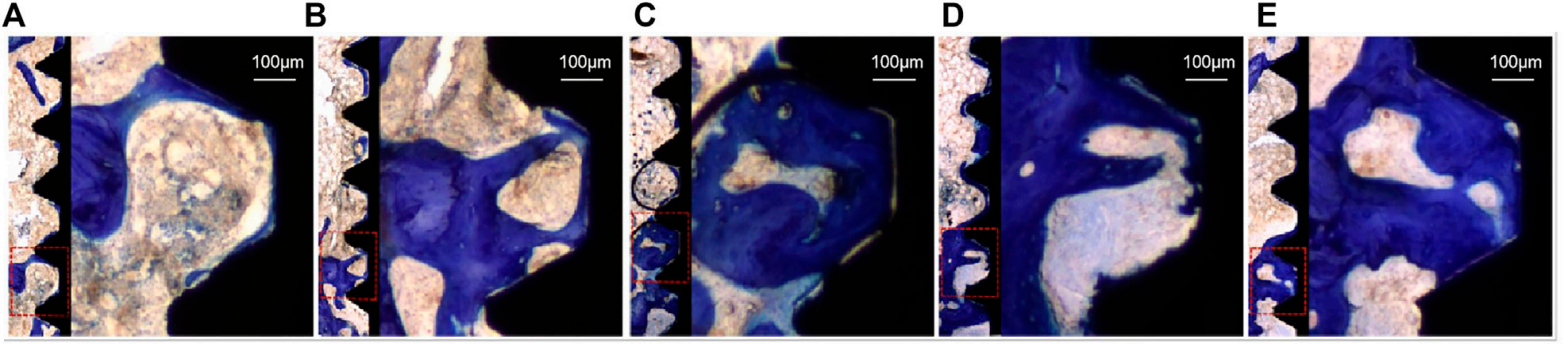
Rat femur toluidine blue staining, dark blue part represents new bone tissue. **(A)**: PT group; **(B)**: NT group; **(C)**: WNT group; **(D)**: KNT-CG group; **(E)**: KWNT-CG group.

Toluidine blue tissue staining of the samples from each group after 4 weeks is shown in Figure 10, in which the black area is the implant thread, white area on the left is the peri-implant bone tissue, and dark blue area is the new bone tissue. Compared to the PT group, the NT, WNT, KNT-CG, and KWNT-CG groups showed significantly more new bone tissue and increased continuity. The PT group had less new bone tissue production, which only existed locally in the implant thread, with discontinuous bone trabeculae and more faults. Compared to the PT group, the NT and WNT groups showed a significant improvement, with an increased amount of continuous new bone. The KNT-CG group showed a further increase in new bone, with only a small number of discontinuities, although the bone trabeculae was not dense. The KWNT-CG group showed dense bone trabeculae at the implant threads, with uniform continuity, full contact between the implant and new bone, and optimal new bone formation on the surface.

## Discussion

The basic building blocks of natural bone are mineralized collagen fibers with a diameter of about 80 nm, while the collagen fibers spiral forward around a Haver’s tube with a thickness of 3 µm. (Gittens et al., 2011; Jeon et al., 2015). Therefore, from a bionic perspective, the surface treatment of implants into micro- and nano-structures more closely resembles the natural bone morphology compared to micron or nano single-level structures. Li et al. (2010) found that the increase in the surface roughness and change in oxygen and fluorine content of titanium implants after HF treatment improved their biocompatibility and thus enhanced the osseointegration ability of implants. Different concentrations of HF solution were used for acid etching to form micron structure and anodic oxidation to form nanotubes with a diameter of around 80 ± 10 nm. The hollow structure facilitates the differentiation of osteoblasts and can be used to accommodate adsorbed drugs and enhance the mechanical embedding ability between the implant surface and bone. Treatment of the implant to form a micron surface also increases its surface area and alters the force on the bone tissue.

Anodization of the implant surface not only changes the surface microstructure, but also affects its hydrophilicity. Hydrophilicity facilitates cell adhesion, proliferation, osteogenesis, and differentiation on the surface by inhibiting immune inflammatory cells and releasing more bioactive factors, further promoting osseointegration (Elias et al., 2008; Günay-Bulutsuz et al., 2018). The use of a combination of nanotubes and micron step structures led to a decrease in the contact angle of the samples, likely because of the greater surface roughness obtained by micro- and nano-binding, thus presenting better hydrophilicity. After loading the surface with drugs, the contact angle did not greatly change, whereas after coating with chitosan and gelatin, the contact angle tended to increase because kaempferol contained fewer hydroxyl groups that could not fully react with the groups in chitosan and gelatin to form hydrogen bonds. However, the hydrophilicity of the experimental group remained higher than that of the control group. Thus, this micro-nano treatment of the implant surface increases hydrophilicity, which is favorable for osteogenesis.

In addition, Filovál found (Elena et al., 2014) that the micro-nanocomposite structure promoted cellular ground attachment growth, proliferation, and differentiation. In our study, the cell survival rate of the NT and WNT groups increased over 7 days, which is consistent with the results of Filová1 showing that the micro-nanostructures promoted cell proliferation. In contrast, the cell survival rate in the KNT and KWNT groups decreased significantly with increasing kaempferol release in the first 3 days. This may be because of the rapid drug release; the cell survival rate gradually stabilized after 3 days with complete release of the drug. Both chitosan and gelatin show good biocompatibility and can be degraded and absorbed by organisms, and chitosan exhibits slow and controlled release (Bastami et al., 2017; Wu et al., 2020). To alleviate reduced cell proliferation caused by rapid drug release, we uniformly coated the surface of titanium sheets loaded with kaempferol in micro-nanomorphic form and constructed KNT-CG and KWNT-CG groups, which did not significantly inhibit cell growth, slowed the drug release rate, increased the release time, increased drug release, and facilitated osseointegration, with the KWNT-CG group showing more obvious advantages. The vacuum method was used to load kaempferol into the nanotubes, eliminating the influence of other gases in the air. This approach also avoids the toxic side effects of systemic administration and increases local drug action.

Nowak et al. (2017) Suggested that oral kaempferol prevents OVX-induced bone loss by inhibiting bone resorption, and Kumar et al. (2012) found that oral kaempferol increases the stimulatory effect on osteoblasts, thereby increasing the bone anabolic effect. To verify the osteogenic effect of kaempferol, we established a rat OP model for *in vivo* experiments. Analysis of the femurs showed that new bone was generated at the implant threads in all experimental groups. The number and thickness of bone trabeculae increased and microstructure of the bone trabeculae was significantly improved. Bone trabeculae at the implant threads in the KWNT-CG group were dense, uniform, and continuous, and the implant was in full contact with new bone, showing optimal new bone formation on the surface. The number and thickness of bone trabeculae increased in the experimental group and the microstructure of bone trabeculae was significantly improved. These results suggest that the combination of micro-nano structures and kaempferol had an anti-osteoporotic effect. The Tb.N, Tb.Th, and BV/TV values increased and Tb. Sp value decreased in all experimental groups, indicating that bone anabolism was greater than bone catabolism, and the combination of the micro-nano structures and kaempferol had anti-OP effect. This is consistent with the results of drastic injury experiments on restoring trabecular structures in rats, confirming the role of kaempferol (Adhikary et al., 2018).

Therefore, the micro- and nano-structures on the implant surface may have promoted osteogenesis and increased the loadable drug volume, but increased drug release increased cytotoxicity and decreased cell proliferation. The surface membrane coverage enabled slow and long-term drug release, mitigating the cytotoxic effects. However, kaempferol loaded on the implant surface has an osteogenic effect, and long-term slow release is beneficial for reducing local OP. The dual effect of the implant micro-nanostructure and kaempferol promoted early osseointegration of the implant. The osteogenic effect of kaempferol may also be related to the induction of osteogenesis by effectors downstream of the mTOR signaling pathway (Zhao et al., 2019). In addition, Trivedi found (Trivedi et al., 2008)that OVX rats given oral kaempferol showed significantly higher bone mineral density and lower serum ALP in the treated trabeculae. Meanwhile, in line with Trivedi’s study, both Ashish (Sharma and Nam, 2019)and (Xie et al., 2021) found in cellular experiments that kaempferol promotes osteogenesis by increasing the activity of ALP and the expression of osteogenic factors Runx2 and osterix.

In conclusion, kaempferol combined with micro-nano-implants promoted the osseointegration of implants and surrounding bone tissue to some extent by constructing TiO_2_ nanotubes and micron structures on the surface of pure titanium flakes loaded with kaempferol under vacuum conditions and coated with a chitosan-gelatin composite film. However, the long-term stability of the micro-nanomorphs loaded with kaempferol is unclear, as we only investigated the early osseointegration for 4 weeks. Long-term properties should be further evaluated.

## Data Availability

The original contributions presented in the study are included in the article/Supplementary Material, further inquiries can be directed to the corresponding author.
